# Usefulness of Combined Measurement of Surfactant Protein D, Thrombin–Antithrombin III Complex, D-Dimer, and Plasmin–α2 Plasmin Inhibitor Complex in Acute Exacerbation of Interstitial Lung Disease: A Retrospective Cohort Study

**DOI:** 10.3390/jcm13082427

**Published:** 2024-04-21

**Authors:** Yuichiro Takeshita, Masako To, Yusuke Kurosawa, Naho Furusho, Toru Kinouchi, Kenji Tsushima, Yuji Tada, Yasuo To, Seiichiro Sakao

**Affiliations:** 1 Department of Pulmonary Medicine, International University of Health and Welfare Narita Hospital, 852 Hatakeda, Narita 286-8520, Japan; 2 Department of Laboratory Medicine, Dokkyo Medical University, Saitama Medical Center, 2-1-50 Minami-Koshigaya, Koshigaya 343-8555, Japan; 3 Division of Respiratory Medicine, Department of Internal Medicine, Nihon University School of Medicine, 30-1 Oyaguchi Kamicho, Tokyo 173-8610, Japan

**Keywords:** coagulopathy, D-dimer, plasmin–α2 plasmin inhibitor complex, pulmonary surfactant protein D, thrombin–antithrombin III complex

## Abstract

**Background/Objectives**: The coagulation cascade due to tissue damage is considered to be one of the causes of poor prognostic outcomes in patients with acute exacerbations of interstitial lung disease (AE-ILD). This study aimed to confirm coagulopathy in AE-ILD by evaluating the differences in the clinical characteristics of coagulation/fibrinolysis markers between stable ILD and AE-ILD. **Methods**: Overall, 81 patients were enrolled in this retrospective study and categorized into the following two groups: a chronic ILD group comprising 63 outpatients and an acute ILD group comprising 18 inpatients diagnosed with AE-ILD. Serum markers, including thrombin–antithrombin III complex (TAT), D-dimer, plasmin–α2 plasmin inhibitor complex (PIC), and surfactant protein D (SP-D), were compared between the groups. **Results**: Among the 18 patients with acute ILD, 17 did not meet the International Society of Thrombosis and Hemostasis scoring system for disseminated intravascular coagulation. In acute ILD, the SP-D levels were statistically significantly positively correlated with TAT, D-dimer, and PIC levels, while the Krebs von den Lungen 6 (KL-6) levels showed no correlation with any of these coagulation/fibrinolytic markers. A positive correlation was observed between SP-D levels and TAT, D-dimer, and PIC levels in acute ILD. Serum TAT, D-dimer, and PIC all showed good area under the receiver operating characteristic (ROC) curve (AUC) values in ROC analysis for the diagnosis of acute ILD. **Conclusions**: In the clinical setting of AE-ILD, it may be important to focus not only on alveolar damage markers such as SP-D but also on coagulation/fibrinolytic markers including TAT, D-dimer, and PIC.

## 1. Introduction

Numerous studies have reported that acute exacerbation of interstitial lung disease (AE-ILD) is a life-threatening event [1,2]. AE-ILD can cause idiopathic pulmonary fibrosis (IPF) or non-IPF [1]. The 1-month mortality rate of AE-IPF ranges from 37% to 53%, and the 3-month mortality rate ranges from 63.8% to 73.7% [1]. ILD, other than IPF, causes acute exacerbations and has a mortality rate as high as that of AE-IPF. Similar to AE-IPF, there is a high risk of acute exacerbation of chronic hypersensitivity pneumonitis (AE-CHP), demonstrated by the overall mortality rate of AE-CHP ranging from 75% to 100%, whereas the AE-related mortality rate of other types of ILD ranges from 34% to 83% [1]. Therefore, patients with AE-ILD have a poor prognosis, regardless of whether it is IPF or non-IPF.

The crosstalk between inflammation and coagulation is important in various pathologies, including respiratory diseases [3]. Hypercoagulation plays a crucial role in fibroproliferative lung diseases. Once tissue factor, activated by tissue injury and inflammation, induces the coagulation pathway, thrombin production, which converts fibrinogen to fibrin, leads to hypercoagulation. Thrombin has a high affinity for protease-activated receptors (i.e., G protein-coupled receptors), which play crucial roles in mediating the interplay between coagulation and inflammation and between tissue repair and fibrosis [3,4]. Furthermore, patients with IPF in such a prothrombotic state appear to have a more severe disease at presentation and an increased risk of death [4,5,6].

The pathology of AE-ILD is considered to be secondary to the activation of coagulation cascades due to tissue damage, which may cause diffuse alveolar damage [2,7,8]. Activated intravascular coagulation disturbance occurs in AE-IPF due to significantly elevated levels of plasma fibrin degradation product, D-dimer, and thrombin–antithrombin III complex (TAT) [7,9]. Moreover, increased fibrin deposition in the alveolar space and increased levels of TAT in the bronchoalveolar lavage fluid are characteristic histological features of IPF [10]. These findings suggest that coagulation/fibrinolysis markers, including TAT, are associated with AE-ILD. However, only a limited number of previous AE-ILD-related reports have focused on coagulation/fibrinolysis markers.

AE-ILD is similar in pathophysiology to acute respiratory distress syndrome (ARDS), characterized by alveolar epithelial damage and coagulopathy [11]. In ARDS, pro-inflammatory stimuli affect the coagulation fibrinolytic system mechanism, inducing epithelial and endothelial damage to the lungs [11]. Therefore, it is important to focus not only on the alveolar epithelial injury markers, including KL-6 and SP-D, but also on the coagulation fibrinolytic system markers, including TAT, D-dimer, and PIC, in the clinical practice of AE-ILD [12,13]. However, there has been no previous report showing the relationship between alveolar epithelial injury markers and coagulation/fibrinolytic markers in AE-ILD using clinical data.

Therefore, this study aimed to deepen our understanding of coagulopathy in AE-ILD by investigating the differences in the characteristics of coagulation/fibrinolysis markers between stable ILD and AE-ILD. Additionally, we aimed to investigate how Krebs von den Lungen 6 (KL-6) and pulmonary surfactant protein D (SP-D), which are known as ILD biomarkers, are involved with the coagulopathy markers [12,13].

## 2. Materials and Methods

### 2.1. Study Design and Patients

All study procedures were conducted in accordance with the standards of the Ethical Review Board of the International University of Health and Welfare (approval number 20-Nr-101 [22 February 2021]) and conformed to the 1964 Declaration of Helsinki and its later amendments or comparable ethical standards. The ethics committee waived the requirement for informed consent because this was a retrospective analysis limited to pre-existing data collected as part of the standard of care by respiratory physicians. Data were anonymized, and patient privacy was protected.

This single-center retrospective study evaluated 109 patients with stable ILD and AE-ILD admitted to the International University of Health and Welfare Narita Hospital (the definitions of stable ILD and AE-ILD are given below). Patients with stable ILD were selected from 86 outpatients who visited in February 2023, whereas those with AE-ILD were selected from 23 inpatients hospitalized between April 2022 and March 2023.

Outpatients diagnosed with IPF, unclassified ILD, idiopathic nonspecific interstitial pneumonia (iNSIP), connective tissue disease-associated interstitial lung disease (CTD-ILD), or CHP were defined as having stable ILD based on medical records and laboratory, high-resolution chest computed tomography (HRCT), and pathologic findings. Among the 86 patients with stable ILD, 5 without laboratory data, 3 with malignancy, 6 treated with >10 mg prednisolone, and 4 without pulmonary function tests were excluded from this study. Based on the interstitial lung disease–gender–age–lung physiology (ILD-GAP) model, known as ILD severity classification, the ILD-GAP index of each patient was calculated [14]. Patients with an ILD-GAP index of ≥0 were selected. The severity classification of the ILD-GAP model in patients with stable ILD was as follows: stage I, 17.5% (*n* = 11); stage II, 52.4% (*n* = 33); stage III, 20.6% (*n* = 13); and stage IV, 9.5% (*n* = 6). Therefore, a total of 63 outpatients with stable ILD were enrolled in this study.

**Figure 1:**
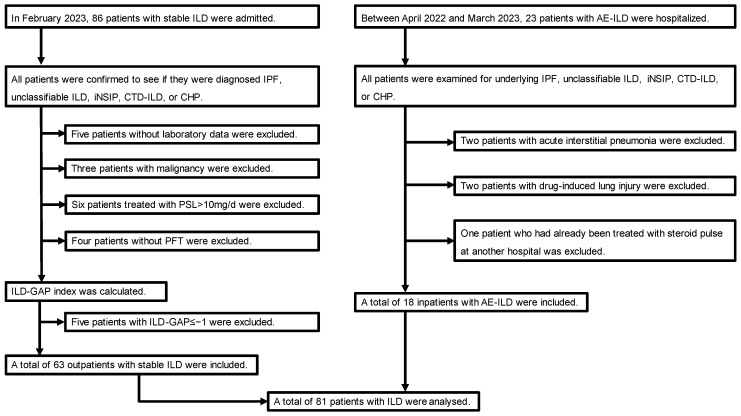
Flowchart of the study population. AE-ILD, acute exacerbation of interstitial lung disease; CHP, chronic hypersensitivity pneumonitis; CTD-ILD, connective tissue disease-associated interstitial lung disease; ILD, interstitial lung disease; ILD-GAP, interstitial lung disease–gender–age–lung physiology; iNSIP, idiopathic nonspecific interstitial pneumonia; IPF, idiopathic pulmonary fibrosis; PFT, pulmonary function test; and PSL, prednisolone.

Among the 23 inpatients with AE-ILD, 4 without underlying IPF, unclassifiable ILD, iNSIP, CTD-ILD, or CHP were excluded from this study (i.e., 2 patients with acute interstitial pneumonia and 2 patients with drug-induced lung injury). Furthermore, one patient who had already been treated with a corticosteroid pulse at another hospital was excluded. Thus, 18 patients with AE-ILD were enrolled in this study. Consequently, 81 patients with chronic or acute ILD were enrolled in this study (Figure 1).

### 2.2. Definition of AE-ILD

To match the background of the stable ILD cohort, the AE-ILD cohort was restricted to patients with underlying IPF, unclassifiable ILD, iNSIP, CTD-ILD, or CHP. The diagnosis of AE-ILD was modified using the revised definition and diagnostic criteria for AE-IPF proposed by an International Working Group Report as follows: (1) previous or concurrent diagnosis of ILD; (2) acute worsening or development of dyspnea, typically <1 month in duration; (3) CT with new bilateral ground–glass opacity and/or consolidation superimposed on a background pattern consistent with ILD; and (4) deterioration not fully explained by cardiac failure or fluid overload [15]. All patients with AE-ILD received at least one course of steroid pulse therapy (methylprednisolone, 1000 mg/day for 3 days/course).

### 2.3. Definition of Disseminated Intravascular Coagulation (DIC)

A patient with a score of ≥5 was categorized as having overt DIC [16,17]. The International Society of Thrombosis and Hemostasis (ISTH) scoring system for DIC, shown in Table 1, was calculated based on laboratory test results obtained within 3 days of the corticosteroid pulse therapy.

### 2.4. Data Collection

All clinical data related to this study were collected from patients’ electronic medical records from 1 May to 30 September 2023. The pulmonary function test and chest HRCT results of patients with stable ILD were extracted from those most recently performed after 2020, and the baseline treatment and laboratory test results performed in February 2023 were extracted. AE-ILD laboratory test results were extracted from those obtained within 3 days of the initiation of steroid pulse therapy, and chest HRCT findings were extracted from the time of admission to the initiation of steroid pulse therapy.

### 2.5. Statistical Analysis

Summary statistics were calculated using the mean ± standard deviation (SD), frequency distributions, or proportions of baseline variables. We first compared the mean ± SD values and quartiles between the two groups for continuous variables. Subsequently, the Kolmogorov–Smirnov (two-sided) and Shapiro–Wilk tests were used to test normality, after which homoscedasticity was determined using the F-test. Next, Welch’s *t*-test and Mann–Whitney *U* test were performed according to the data distribution. We first compared the mean ± SD values and quartiles between the two groups for continuous variables, such as age and body mass index (BMI). Fisher’s exact test was used to determine the significance of the differences between groups. Cut-off values were also evaluated using receiver operating characteristic (ROC) curve analysis and the area under the ROC curve (AUC). Higher AUC values demonstrated superior discriminatory ability as follows: excellent discrimination, 0.9 ≤ AUC; good discrimination, 0.80 ≤ AUC < 0.90; fair discrimination, 0.70 ≤ AUC < 0.80; and poor discrimination, AUC < 0.70. For a diagnostic test to be meaningful, the AUC has to be >0.5 [18,19]. The correlation between different parameters was analyzed using Spearman’s correlation test. The results of Spearman’s correlation tests were evaluated as follows: strong (*r* = 0.7–1.0), moderate (*r* = 0.5–0.7), or low (*r* = 0.3–0.5) [20]. Finally, all statistical analyses were conducted using EZR (Saitama Medical Center, Jichi Medical University, Saitama, Japan), a graphical user interface for R, and a modified version of the R commander designed to add statistical functions that are frequently used in biostatistics [21]. Statistical significance was set at *p* < 0.05.

## 3. Results

### 3.1. Patient Background

Table 2 shows the clinical characteristics of the 81 patients enrolled in this study by comparing the stable ILD (*n* = 63) and AE-ILD (*n* = 18) groups. Univariate analysis showed no significant difference between these two groups, except for BMI and the frequency of anticoagulant drugs as a baseline treatment.

### 3.2. Comparison of Laboratory Findings between the Stable ILD and AE-ILD Groups

Table 3 shows the univariate analysis comparing laboratory findings between the stable ILD and AE-ILD groups (the results regarding the surfactant protein D [SP-D], TAT, D-dimer, and plasmin–α2 plasmin inhibitor complex [PIC] levels are described below). C-reactive protein, lactate dehydrogenase, interleukin-6 (IL-6), prothrombin time international normalized ratio (PT-INR), and fibrinogen levels in the AE-ILD group were significantly higher than those in the stable ILD group.

### 3.3. Characteristics of Patients with AE-ILD

Table 4 shows the characteristics of the 18 patients with AE-ILD. Among these 18 patients, only one satisfied the DIC criteria.

All 18 patients were treated with 3 days of corticosteroid pulse therapy, and combination therapies, including tacrolimus and/or direct hemoperfusion using a polymyxin B-immobilized fiber column, were performed according to the general condition of each patient. Each patient’s oxygen device was described based on the worst respiratory condition during hospitalization (no oxygen device was administered to Patient 3; however, they had a chief complaint of dyspnea on exertion).

### 3.4. Relationships between Alveolar Epithelial Injury and Coagulopathy Markers in AE-ILD

**Figure 2:**
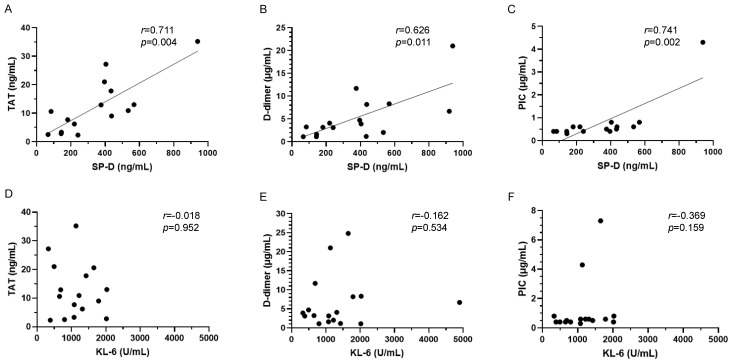
Correlation between alveolar epithelial injury and coagulopathy markers in AE-ILD. (**A**) SP-D and TAT, (**B**) SP-D and D-dimer, (**C**) SP-D and PIC, (**D**) KL-6 and TAT, (**E**) KL-6 and D-dimer, (**F**) KL-6 and PIC. AE-ILD, acute exacerbation of interstitial lung disease; KL-6, Krebs von den Lungen 6; PIC, plasmin–α2 plasmin inhibitor complex; SP-D, pulmonary surfactant protein D; and TAT, thrombin–antithrombin III complex.

To evaluate the relationship between AE-ILD and coagulopathy, we selected SP-D and KL-6 as indicators of alveolar epithelial injury markers, and TAT, D-dimer, and PIC as indicators of coagulopathy. Figure 2A–C show the correlation between SP-D and coagulation/fibrinolysis markers in patients with AE-ILD. TAT, D-dimer, and PIC levels were significantly positively correlated with SP-D levels. Specifically, TAT and PIC levels showed strong correlations with SP-D levels. No significant correlations were observed between SP-D and other coagulation/fibrinolysis markers. On the other hand, KL-6 showed no correlation with any of the coagulation/fibrinolysis markers (Figure 2D–F).

### 3.5. Relationships between Alveolar Epithelial Injury and Coagulopathy Markers in Stable ILD

**Figure 3:**
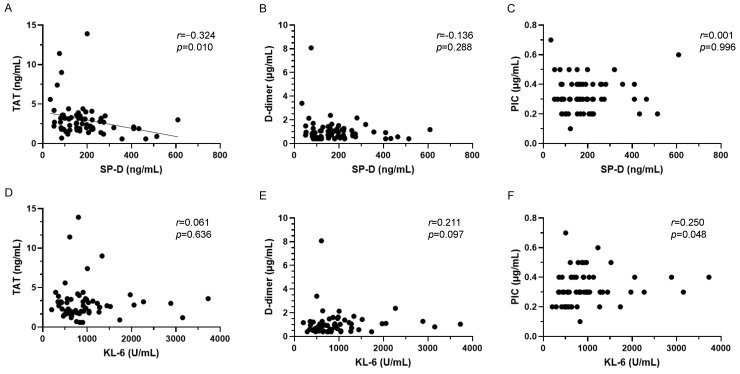
Correlation between SP-D and coagulation/fibrinolysis markers in stable ILD. (**A**) SP-D and TAT, (**B**) SP-D and D-dimer, (**C**) SP-D and PIC, (**D**) KL-6 and TAT, (**E**) KL-6 and D-dimer, (**F**) KL-6 and PIC. ILD, interstitial lung disease; KL-6, Krebs von den Lungen 6; PIC, plasmin–α2 plasmin inhibitor complex; SP-D, pulmonary surfactant protein D; and TAT, thrombin–antithrombin III complex.

Similar to AE-ILD, the correlation between alveolar epithelial injury markers, including SP-D and KL-6, and coagulopathy markers, including TAT, D-dimer, and PIC, was analyzed in stable ILD (Figure 3). Unlike AE-ILD, SP-D and TAT showed a weak negative correlation in stable ILD. No significant correlation was observed between SP-D and other coagulation/fibrinolysis markers. KL-6 showed no correlation with any coagulation/fibrinolytic markers.

### 3.6. AE-ILD Diagnostic Accuracy of Alveolar Epithelial Injury and Coagulopathy Markers

**Figure 4:**
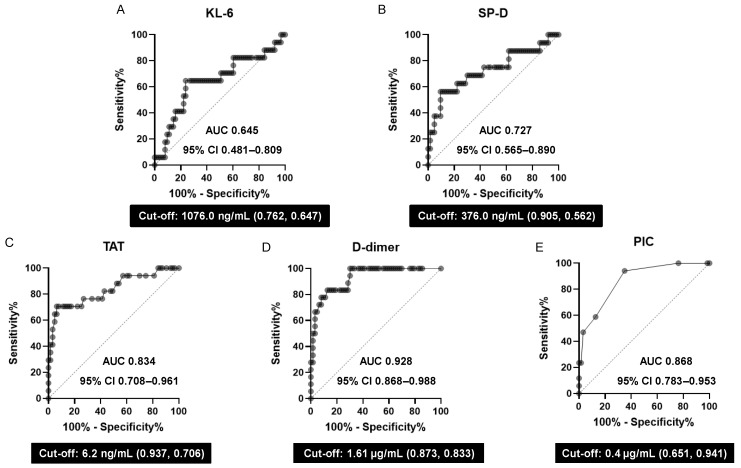
AE-ILD diagnostic accuracy of alveolar epithelial injury and coagulopathy markers. The ROC curves for the highest AUC values and the cut-off (specificity and sensitivity) values, AUCs, and 95% CIs for each biomarker, (**A**) KL-6, (**B**) SP-D, (**C**) TAT, (**D**) D-dimer, and (**E**) PIC, are shown. AE-ILD, acute exacerbation of interstitial lung disease; AUC, area under the ROC curve; CI, confidence interval; KL-6, Krebs von den Lungen 6; PIC, plasmin–α2 plasmin inhibitor complex; ROC, receiver operating characteristic; SP-D, pulmonary surfactant protein D; and TAT, thrombin–antithrombin III complex.

To evaluate the diagnostic accuracy of alveolar epithelial injury markers and coagulopathy markers for AE-ILD, we performed ROC analysis of KL-6, SP-D, TAT, D-dimer, and PIC, calculating the cut-off values for each of the five biomarkers (Figure 4). The AUC value of the D-dimer was 0.928 (95% confidence interval [CI]: 0.868–0.988), indicating excellent discrimination. The AUC values of TAT and PIC were 0.834 (95% CI: 0.708–0.961) and 0.868 (95% CI: 0.783–0.953), respectively, indicating good discrimination. The AUC value of SP-D was 0.727 (95% CI: 0.565–0.890), indicating fair discrimination. The AUC value of KL-6 was 0.645 (95% CI: 0.481–0.809), indicating poor discrimination.

## 4. Discussion

Our study has several major findings. First, among the 18 patients with AE-ILD, all except 1 patient did not satisfy the ISTH scoring system for DIC. Second, ROC analysis demonstrated that TAT, D-dimer, and PIC all showed good AUC values, suggesting useful markers for diagnosing AE-ILD. Third, in univariate analysis, the SP-D values in the AE-ILD group were significantly higher than those of stable ILD, while the KL-6 values showed no significant difference between the two groups. Fourth, the SP-D values in the AE-ILD group showed a positive correlation with TAT, D-dimer, and PIC values, whereas the stable ILD group showed no positive correlation among them. Fifth, KL-6 levels showed no correlation with coagulation/fibrinolytic markers in either the stable ILD or AE-ILD groups.

DIC is an acquired syndrome characterized by systemic intravascular activation of coagulation that can be caused by infectious and non-infectious insults [22]. The main pathophysiological mechanisms of DIC are explained by the production of large amounts of tissue factor due to the release of inflammatory cytokines, resulting in insufficient regulation of anticoagulation pathways and inhibition of fibrinolysis [22]. Consequently, endothelial dysfunction and microvascular thrombosis occur, which can seriously impact patient prognosis as this causes multiple organ failure [22,23]. In our study, only 5.6% (1/18 patients) of AE-ILD cases met the DIC diagnostic criteria. Although AE-ILD is similar to DIC in that it causes coagulopathy, it is generally believed that it is not necessarily a systemic pathology like DIC but rather a localized coagulopathy, as exemplified by diffuse alveolar damage [22,23,24]. Therefore, it may be challenging to apply the DIC scoring system to AE-ILD.

Previous reports have suggested that AE-ILD is associated with coagulopathy and that D-dimer is a useful predictive marker for AE-ILD [7]. Another report has also mentioned the association between coagulation factors and AE-ILD [25]. In this study, TAT, D-dimer, and PIC showed good AUC values in the ROC analysis for AE-ILD. To support the diagnosis of AE-ILD, it may be important to measure not only D-dimer but also other coagulation fibrinolytic markers, including TAT and PIC.

SP-D is a lipoprotein complex synthesized and secreted from type II alveolar epithelial cells that constitutes a liquid layer on the alveolar epithelium. Elevated serum SP-D levels are believed to be the result of alveolar epithelial injury and the breakdown and accumulation of type II alveolar epithelial cells [26,27]. Multiple reports exist regarding SP-D and AE-IPF, including a report that mentions that SP-D values are higher in patients with AE-IPF than in those with stable disease [28]. Additionally, SP-D has been reported as a poor prognostic factor for AE-IPF [26,27]. Our study also showed in univariate analysis that SP-D was significantly higher in patients with AE-ILD than in those with stable ILD. According to previous reports, SP-D rapidly leaks into blood vessels via capillaries due to alveolar epithelial injury [29,30]. Therefore, the relationship between SP-D and coagulation/fibrinolysis markers in AE-ILD revealed in this study suggests that coagulopathy is related to alveolar epithelial injury in AE-ILD. Although SP-D and TAT were correlated in AE-ILD in this study, it may not be possible to conclude that elevated serum TAT in AE-ILD indicates local coagulation activity in the lungs. However, this study may indicate the importance of evaluating coagulation/fibrinolysis markers when faced with AE-ILD in clinical practice. Moreover, it may be important for reviewing clinical anticoagulant therapy and elucidating the pathogenesis of AE-ILD. Further basic research and large-scale prospective studies are required to support the findings of this study.

The present study demonstrated that KL-6 levels showed no correlation with TAT, D-dimer, or PIC in either group, suggesting that KL-6 may not be suitable for use in the diagnosis of AE-ILD in combination with coagulation/fibrinolytic markers. Unlike SP-D, which leaks directly into the blood via capillaries, KL-6 is released from regenerated type II pneumocytes into the blood via respiratory bronchiolar epithelial cells and basement membranes damaged by recruited inflammatory cells [30,31]. Therefore, the difference in the mechanism of the increase in serum SP-D and KL-6 may have influenced the difference in the relationship with coagulation/fibrinolytic markers.

In our study, the BMI values of the AE-ILD group were significantly lower than those of the stable ILD group, which is consistent with the results of a previous report. According to a previous study of BMI and mortality in patients with AE-IPF, classified as underweight (<18.5 kg/m^2^), low–normal weight (18.5–22.9 kg/m^2^), high–normal weight (23.0–24.9 kg/m^2^), overweight (25.0–29.9 kg/m^2^), and obese group (≥30.0 kg/m^2^), the underweight group showed higher mortality and the obese group showed lower mortality than low-normal weight group [32]. This previous report was researched based on the Japanese Diagnosis Procedure Combination database, including 14,783 patients with AE-IPF [32]. Therefore, the BMI results obtained in this study may be reasonable.

Our study had some limitations. First, this was a single-center, retrospective study. Further data collection is required in the future. Second, the sample size was small. This may be due to the short observation period. The incidence and prevalence of IPF are 2.23 and 10.0 per 100,000 individuals, respectively [33]. The annual incidence of acute exacerbations after IPF diagnosis is approximately 10 per 100 patient-years from the second year onwards [34]. Therefore, larger, long-term observational studies are warranted to validate the study’s findings. Third, univariate analysis demonstrated that the frequency of anticoagulant drugs as a baseline treatment in the AE-ILD group was significantly higher than that of the stable ILD group. A multivariate analysis should have been performed to examine whether the results of this univariate analysis affected the relationship between SP-D and KL-6 and coagulation/fibrinolytic markers. However, we judged the sample size of this study to be too small for multivariate analysis. Therefore, this study was limited to a univariate analysis.

## 5. Conclusions

SP-D, TAT, D-dimer, and PIC levels may be useful diagnostic markers for AE-ILD. Particularly, a combined measurement of SP-D and coagulation fibrinolytic markers, including TAT, D-dimer, and PIC, may be useful in the diagnosis of AE-ILD. Further studies are required to confirm these findings.

## Figures and Tables

**Table 1:** ISTH scoring system for DIC.

Variable	Value	Points
Plt (×10^3^/μL)	>100	0
	50–100	1
	<50	2
PT-INR	<1.3	0
	1.3–1.7	1
	>1.7	2
D-dimer (μg/mL)	<0.4	0
	0.4–4.0	2
	>4.0	3
Fibrinogen (mg/dL)	>100	0
	<100	1

DIC, disseminated intravascular coagulation; ISTH, International Society of Thrombosis and Hemostasis; Plt, platelet; and PT-INR, prothrombin time-international normalized ratio.

**Table 2:** Patient background.

Variables	Stable ILD	AE-ILD	*p*-Value
	(*n* = 63)	(*n* = 18)	
Demographic			
Age (years)	75.0 (72.0, 80.5)	76.0 (74.0, 80.0)	0.645
Male sex (%)	37 (58.7)	13 (72.2)	0.412
BMI (kg/m^2^)	24.48 (22.30, 25.85) (N/A = 14)	20.66 (19.63, 23.85) (N/A = 1)	0.004
Smoking history (%)	36 (63.2) (N/A = 6)	15 (83.3)	0.150
Baseline ILD			
IPF (%)	37 (58.7)	14 (77.8)	0.174
Unclassifiable ILD (%)	8 (12.7)	0 (0.0)	0.189
iNSIP (%)	8 (12.7)	2 (11.1)	>0.999
CTD-ILD (%)	7 (11.1)	1 (5.6)	0.677
CHP (%)	3 (4.8)	1 (5.6)	>0.999
Baseline treatment			
Corticosteroids (%)	22 (34.9)	3 (16.7)	0.162
Immunosuppressants (%)	1 (1.6)	2 (11.1)	0.123
Antifibrosis drugs (%)	36 (57.1)	9 (50.0)	0.603
Anticoagulant drugs (%)	3 (4.8)	6 (33.3)	0.003
Home oxygen therapy (%)	14 (22.2)	7 (38.9)	0.221

AE-ILD, acute exacerbation of interstitial lung disease; BMI, body mass index; CHP, chronic hypersensitivity pneumonitis; CTD-ILD, connective tissue disease-associated interstitial lung disease; ILD, interstitial lung disease; iNSIP, idiopathic nonspecific interstitial pneumonia; and IPF, idiopathic pulmonary fibrosis; N/A, not applicable.

**Table 3:** Comparison of laboratory findings between the stable ILD and AE-ILD groups.

Laboratory Findings	Stable ILD	AE-ILD	*p*-Value
	(*n* = 63)	(*n* = 18)	
CRP (mg/dL)	0.16 (0.11, 0.63)	12.45 (5.94, 17.27)	<0.001
LDH (U/L)	229 (196, 262)	387 (272, 478) (N/A = 1)	<0.001
PCT (ng/mL)	0.05 (0.03, 0.06)	0.16 (0.12, 0.47) (N/A = 1)	<0.001
IL-6 (pg/mL)	3.7 (2.3, 7.5)	28.5 (6.8, 125.7)	<0.001
KL-6 (U/mL)	803 (562, 1043)	1132 (687, 1651) (N/A = 1)	0.068
SP-D (ng/mL)	167 (102, 224)	386 (172, 461)	0.005
Plt (×10^3^/μL)	221 (181, 262)	217 (204, 276)	0.725
PT-INR (s)	0.94 (0.92, 0.97)	1.07 (1.00, 1.13)	<0.001
Fibrinogen (mg/dL)	353 (313, 404)	564 (470, 693) (N/A = 1)	<0.001
TAT (ng/mL)	2.5 (1.9, 3.4)	10.6 (3.3, 17.8)	<0.001
D-dimer (μg/mL)	0.88 (0.57, 1.20)	3.57 (2.07, 7.81)	<0.001
PIC (μg/mL)	0.3 (0.3, 0.4)	0.5 (0.4, 0.6)	<0.001

AE-ILD, acute exacerbation of interstitial lung disease; CRP, C-reactive protein; IL-6, interleukin 6; ILD, interstitial lung disease; KL-6, Krebs von den Lungen 6; LDH, lactate dehydrogenase; PCT, procalcitonin; Plt, platelet; PT-INR, prothrombin time-international normalized ratio; PIC, plasmin–α2 plasmin inhibitor complex; SP-D, pulmonary surfactant protein D; and TAT, thrombin–antithrombin III complex.

**Table 4:** Characteristics of patients with AE-ILD.

P	Baseline				Hospitalization					
	Age (y)	Sex	ILD	ILD Treatment	DIC Score	Code Status	Treatment in Combination with Steroid Pulse	Oxygen Device	Hospitalization (Days)	Outcome
1	81	M	CHP	None	3	DNI	–	HFOT	22	Survivor
2	72	M	IPF	None	2	Full	–	HFOT	12	
3	79	F	IPF	Nintedanib	0	Full	–	None	10	
4	83	F	iNSIP	None	3	DNI	–	HFOT	23	
5	75	M	IPF	None	3	DNI	Tacrolimus	HFOT	35	
6	76	F	iNSIP	None	2	DNI	Tacrolimus	NPPV	36	
7	76	M	IPF	Nintedanib	2	DNI	PMX-DHP	HFOT	20	
8	74	F	IPF	Pirfenidone + PSL (9 mg/d)	4	DNI	Tacrolimus	HFOT	19	
9	73	M	IPF	Nintedanib	2	Full	–	COT	11	
10	71	M	IPF	Pirfenidone	3	DNI	Tacrolimus + PMX-DHP	HFOT	40	Non-survivor
11	80	M	IPF	Nintedanib	3	DNI	Tacrolimus + PMX-DHP	HFOT	23	
12	80	F	IPF	Tacrolimus	3	DNI	Tacrolimus + PMX-DHP	HFOT	16	
13	75	M	IPF	Nintedanib	5	DNI	Tacrolimus + PMX-DHP	NPPV	38	
14	74	M	IPF	None	2	Full	–	MV	33	
15	80	M	CTD-ILD	Azathioprine + PSL (5 mg/d)	2	DNI	Tacrolimus + PMX-DHP	HFOT	8	
16	82	M	IPF	Nintedanib	2	DNI	Tacrolimus	HFOT	27	
17	79	M	IPF	None	3	Full	Tacrolimus + PMX-DHP	MV	23	
18	56	M	IPF	Pirfenidone + PSL (20 mg)	3	DNI	Tacrolimus + PMX-DHP	NPPV	11	

AE-ILD, acute exacerbation of interstitial lung disease; CHP, chronic hypersensitivity pneumonitis; COT, conventional oxygen therapy; CTD-ILD, connective tissue disease-associated interstitial lung disease; DIC, disseminated intravascular coagulation; DNI, not intubated; F, female; HFOT, high-flow oxygen therapy; ILD, interstitial lung disease; iNSIP, idiopathic nonspecific interstitial pneumonia; IPF, idiopathic pulmonary fibrosis; M, male; MV, mechanical ventilation; NPPV, noninvasive positive pressure ventilation; P, patient; PMX-DHP, polymyxin B-immobilized fiber column; and PSL, prednisolone.

## Data Availability

The data and materials are available from the corresponding authors upon reasonable request.
